# Fostering effective vocabulary retention among primary school students: a case study

**DOI:** 10.3389/fpsyg.2025.1594620

**Published:** 2025-11-14

**Authors:** Damla Sahin, Mehmet Ali Yavuz

**Affiliations:** Department of English Language Teaching, Cyprus International University, Nicosia, Cyprus

**Keywords:** vocabulary learning, young learners, cognitive load, working memory, meaning-first instruction, form-first instruction, lexical access

## Abstract

**Introduction:**

Numerous studies on vocabulary acquisition have prioritized form-first instruction, focusing on spelling and pronunciation before meaning. However, cognitive theories suggest that introducing meaning first may enhance vocabulary retention by engaging deeper semantic and conceptual processing.

**Methods:**

This quasi-experimental study explored the effects of meaning-first versus form-first vocabulary instruction on bilingual primary school students in Cyprus. Over a 12-week period, 57 fourth-grade students were divided into two groups: one receiving meaning-first instruction and the other form-first instruction. The Self-Report Vocabulary Mastery Scale (SRVMS) was used to assess both word knowledge and contextual application.

**Results:**

Findings revealed that while both groups improved, the meaning-first group significantly outperformed the form-first group in vocabulary acquisition and sentence-level usage. Statistical analysis showed a large effect size (Cohen's *d* = 1.37), indicating a strong advantage for meaning-first instruction in fostering lexical encoding and retrieval.

**Discussion:**

The results align with theories of cognitive load, depth of processing, and working memory, offering practical implications for inclusive and sustainable language education.

## 1 Introduction

Vocabulary is the bridge between language learning and real communication. For young learners, building a strong vocabulary helps unlock all areas of learning whether it's understanding what they hear in class, reading with confidence, participating in discussions, or expressing their own ideas in writing. In fact, vocabulary plays a foundational role in the development of the four key language skills: listening, speaking, reading, and writing (Beck et al., 2002; Nation, 2013). Without access to a broad range of words, children can find it difficult to follow lessons, make meaning from texts, or articulate their thoughts. Over time, this can affect not only their academic progress but also their sense of confidence and connection in the classroom.

In bilingual school environments, such as those in Cyprus, vocabulary development becomes even more central. Many students grow up navigating two languages from an early age using one at home and another in school. While this can strengthen cognitive flexibility and awareness of language structure, it can also create moments of disconnection, especially when classroom instruction does not align with learners' prior language experience. For this reason, vocabulary instruction in bilingual settings must do more than teach word lists it needs to engage students meaningfully and provide cognitive support that allows them to retain and use new words confidently. Traditionally, vocabulary teaching has followed a form-first approach, where the structural aspects of words such as pronunciation, spelling, or part of speech are introduced before learners understand the meaning. This model draws from structuralist language pedagogy and is supported by research showing that attention to form can aid recognition and accuracy (Laufer, 2005; Ellis and Beaton, 1993). However, while this approach can be helpful for some learners, it may not always lead to long-term retention or meaningful use, particularly in early bilingual education, where learners benefit from rich, contextual input rather than isolated linguistic forms.

An alternative method, meaning-first instruction, has gained attention over the past decade. This approach aims to create cognitive gaps in learners' minds, prompting them to actively search for the correct target word. Such engagement can lead to improved long-term vocabulary retention. This approach is grounded in well-established cognitive and psycholinguistic theories, including depth of processing (Craik and Lockhart, 1972), working memory (Baddeley, 2003), and dual coding theory (Paivio, 1991). These theories suggest that when learners first engage with what a word means especially in a situation that feels real or familiar they create stronger and more accessible memory traces. Meaning-first instruction can also make learning easier for young learners by linking new words to concepts they already know. This reduces mental strain, as it avoids overwhelming them with unfamiliar sounds and spellings (Sweller, 1994).

Recent findings in neurolinguistics also support the effectiveness of this approach. Studies show that when learners engage with meaning, it activates important areas of the brain responsible for understanding and storing language like the left inferior frontal gyrus and the posterior temporal cortex (Pulvermüller, 2013). This means that meaning-first instruction may not just seem easier for learners it might actually work in harmony with the brain's natural way of processing language.

Despite growing interest in these approaches, there is still a shortage of classroom-based research comparing form-first and meaning-first instruction especially with young bilingual learners. Most existing studies have focused on older, monolingual students or were conducted in controlled environments that don't reflect the realities of everyday classrooms. As a result, teachers working in bilingual primary settings still lack clear, evidence-based guidance on which method works best. This study seeks to fill that gap. Conducted in a bilingual school in Cyprus, it explores how meaning-first and form-first vocabulary instruction affect vocabulary retention and use among Grade 4 students. This study uses a quasi-experimental design, combining insights from cognitive theory with the realities of everyday classroom life. By exploring how students engage with and retain vocabulary in their daily school environment, the research aims to provide practical guidance for teachers, especially those working in diverse and multilingual classrooms, who are looking for inclusive, research-based strategies that directly support learning.

### 1.1 Theoretical framework

When children start learning new words in another language, how those words are introduced makes a big difference in how well they understand, remember, and use them. This is especially true in English-medium classrooms where many learners are exposed to more than one language in their daily lives. In these settings, vocabulary instruction must be informed by how children naturally process and retain language. Many research studies research from cognitive psychology, psycholinguistics, and neuroscience offers valuable insights into why certain instructional methods particularly meaning-based approach may be more effective than others. The Depth of Processing Theory (Craik and Lockhart, 1972) provides an important foundation for understanding vocabulary retention. It suggests that the more deeply learners engage with new information by attaching it to meaning or context the more likely it is to be remembered. This contrasts with surface-level memorization, where a word's form might be repeated without a deeper understanding. In vocabulary learning, depth of processing implies that students will retain new words more effectively when they are introduced through meaningful stories, discussions, or images, rather than through isolated drills. This idea aligns with Baddeley's (2003) model of working memory, which describes how learners actively hold and work with new information as they try to understand it in the moment. For young learners especially those navigating more than one language cognitive overload can happen when they're faced with too much unfamiliar information at once. Meaning-first instruction helps ease this burden by letting learners first connect new words to something meaningful or visual, before focusing on how the word looks or sounds. This approach supports working memory and helps new vocabulary move more smoothly into long-term memory. Cognitive Load Theory (Sweller, 1994) also supports this idea, emphasizing that the way a learning task is structured plays a big role in how well it's processed. When students are overwhelmed by new sounds, spellings, or grammar rules without first grasping the meaning, their brains use up valuable resources managing that confusion. This type of mental effort is called extraneous cognitive load and it can get in the way of real learning. However, if new words are introduced in a way that connects with learners' prior experiences, visual cues, or classroom activities, the learning becomes more intuitive and less demanding. In line with this, Dual Coding Theory (Paivio, 1991) highlights the power of connecting visual and verbal information. When students are exposed to a word through both a spoken explanation and a picture or gesture, they form two mental representations; verbal and visual which make the word easier to remember and to use. This method strengthens memory and increases the chances of using the vocabulary meaningfully in communication. Beyond cognitive perspectives, neuroscientific research reinforces the advantages of meaning-rich instruction. Studies show that areas of the brain involved in semantic processing such as the left inferior frontal gyrus and the posterior temporal cortex are more active when learners focus on understanding a word's meaning rather than relying on only memorization (Pulvermüller, 2013). This suggests that meaning-first methods are not only pedagogically effective but also biologically aligned with how children acquire language. Moreover, psycholinguistic research (e.g., Jenkins and Dixon, 1983; Milton, 2010) consistently finds that words learned in context are more easily retrieved and more accurately used in both spoken and written language. When learners come across new vocabulary in real-life situations instead of isolated word lists, they're more likely to understand and remember the words and actually use them in everyday conversations. This approach is especially helpful for students in multilingual settings, where both their languages and the context around them play a role in how well they retain and use new words. Despite these well-supported theories, there is still a noticeable gap in the empirical literature comparing meaning-first and form-first vocabulary instruction in real classroom contexts particularly in bilingual or multilingual primary school settings. Most studies to date have focused on adult learners or monolingual populations, leaving a lack of practical guidance for educators working with younger students who are developing language skills across two or more languages. Moreover, few studies explore how theoretical principles from cognitive science are applied in structured vocabulary lessons using actual instructional materials. This study aims to fill that gap by comparing how meaning-first and form-first vocabulary instruction affect vocabulary retention and usage among Grade 4 students in a bilingual English-medium school. Rooted in cognitive and psycholinguistic theory and carried out in a real classroom setting, the study offers practical insights for teachers and contributes to the ongoing discussion about inclusive, research-based language teaching. Building on the theoretical foundations discussed above, the study explores how different approaches to vocabulary instruction influence language development in multilingual contexts. To guide this inquiry, the study addresses the following research questions.

1. Does the meaning-first or form-first approach result in differences in vocabulary learning and retention?

a) Is there a significant difference in the pre-test results between both groups?
b) Is there a significant difference in the post-test results between both groups?

## 2 Literature review

Vocabulary development plays a central role in language proficiency, and its instruction must go beyond memorization. Krashen's (2004) Comprehension Hypothesis emphasizes that language acquisition occurs most effectively when learners are exposed to meaningful, understandable input. According to Krashen, vocabulary is best acquired not through rote learning but through rich, contextualized language use that engages the learner's interest and understanding. This aligns with the rationale behind meaning-first instruction, where learners interact with words in context before dissecting their form. Supporting this perspective, Lewis (1993) introduced the Lexical Approach, which argues that language is fundamentally composed of lexical chunks rather than isolated grammar rules. From this view, vocabulary should be taught as patterns and phrases within authentic language use. Lewis advocates for exposing learners to real-world language in use, encouraging acquisition through noticing and internalizing meaningful input. These theoretical foundations support the present study's aim to explore whether meaning-based vocabulary instruction fosters deeper, more durable word knowledge in young bilingual learners.

In recent years, research has increasingly focused on meaning-first instruction, where vocabulary is introduced through rich, meaningful contexts before looking at its structure. This mirrors how children naturally pick up language by linking words to real-life experiences, visuals, and stories. Nation (2022) highlights the value of exposing learners to vocabulary in meaningful settings with repeated encounters, rather than relying on isolated drills. In the same vein, Teng (2021) found that instruction emphasizing meaning over form led to much better retention and more accurate use of vocabulary among young learners. Storytelling has also emerged as a powerful tool for vocabulary development. Reynolds (2023) demonstrated that primary EFL students not only remembered new words better but also used them more confidently when they were embedded in emotionally resonant narratives. This supports the idea that semantic richness and personal connection deepen learners' lexical understanding. In bilingual or multilingual settings, this becomes even more crucial. García et al. (2017) offer a sociocognitive perspective on vocabulary learning, suggesting that bilingual children don't separate their languages into neat compartments they move fluidly between them. Their research supports the use of multilingual language practices and meaning-rich environments to make vocabulary more understandable and relevant. This approach is particularly valuable in diverse educational contexts like Cyprus, where many children grow up hearing and using multiple languages at home, at school, and in the community even if they're not officially recognized as bilingual within the school curriculum. Another perspective comes from Dual Coding Theory (Paivio, 1991) and recent neurocognitive research. Lehmann and Ettinger (2023) showed that when vocabulary is introduced through both verbal and visual formats such as images or stories learners activate multiple memory channels, improving long-term retention. This is consistent with Pulvermüller's (2013) findings, which revealed that meaning-based vocabulary instruction activates deeper brain regions associated with semantic processing.

Although earlier studies laid the groundwork for understanding how vocabulary is learned (Reynolds, 2023; Baddeley, 2003; Sweller, 1994; Ellis and Beaton, 1993), more recent research has provided real-world evidence to support these ideas especially in multilingual classrooms. For example, McKeown et al. (2021) showed that teaching vocabulary through rich, story-based contexts helped learners form stronger mental connections, particularly when visuals or dramatization were used. Still, there's a noticeable gap in research focusing on young learners in bilingual or English-medium schools, especially in regions that are often overlooked. While much of the existing work centers on monolingual learners or adult ESL students, children who grow up using two languages in everyday life have not received as much attention in vocabulary-focused classroom studies. This study helps address that gap by exploring how meaning-first and form-first instruction affect vocabulary learning among Grade 4 students in an English-medium school. In doing so, it adds to ongoing discussions in applied linguistics, education, and cognitive psychology, and offers practical guidance for teaching in linguistically diverse classrooms.

## 3 Research design

This study adopts a quasi-experimental design to evaluate the effects of meaning-first and form-first vocabulary instruction on young learners' word acquisition. Due to institutional constraints, such as intact classroom groupings, random assignment at the individual level was not feasible. Instead, existing classes were used as comparison groups, a common approach in educational settings where true experimental conditions are difficult to implement (Creswell and Creswell, 2018).

The research was conducted at an English-medium primary school in Cyprus, where Grade 4 students were selected as participants. This age group was chosen because they are at a developmental stage where vocabulary instruction shifts from concrete to abstract word learning an important transition supported by prior cognitive developmental research (Anglin et al., 1993; Gathercole et al., 1992). To ensure that all students included in the study had a sufficient level of English to participate meaningfully, the Cambridge English Movers test was administered at the outset. This test, designed for children at the A1 level of the Common European Framework of Reference (CEFR), assesses listening, reading, writing, and speaking skills. It is well-suited to young learners and is widely used in educational settings for placement and diagnostic purposes. On the other hand, the Movers test was not used to measure the outcomes of the study, but solely as a screening tool for participant selection. The test uses a shield scoring system, where students can receive a maximum of 15 shields five for each of the three components (Listening, Reading and Writing, and Speaking). Based on school policy and in consultation with two English language teachers, a benchmark of 10 out of 15 shields was established to determine readiness for the vocabulary intervention. Out of the original 80 students who took the test, 23 students scored below this benchmark and were excluded from the study to ensure a more homogeneous group in terms of language proficiency. This allowed the focus of the research to remain on comparing instructional methods rather than being confounded by wide differences in baseline English skills. The selected students were randomly assigned to two groups (Comparison Group 1 and Comparison Group 2) using a coin flip to eliminate selection bias. The number of participants is presented in the Table 1.

**Table 1:** Number of participants in Comparison Group 1 (CG1) and Group 2 (CG2).

**Groups**	***N***	**Female**	**Male**
Comparison Group 1	29	15	14
Comparison Group 2	28	13	15

A priori power analysis was conducted using G^*^Power 3.1 to determine the minimum required sample size for detecting a medium effect (Cohen's *d* = 0.5) using a two-tailed independent-samples *t*-test, with α = 0.05 and power (1–β) = 0.80. The analysis indicated that a minimum of 54 participants (27 per group) would be sufficient. The actual sample size in this study (*N* = 54) meets this criterion, supporting the statistical adequacy of the design. This justification aligns with sample size recommendations in prior experimental studies on vocabulary instruction (e.g., Teng, 2021; Webb, 2008).

### 3.1 Research instruments

In this study, two research instruments were used to collect data from the learners: the Demographic Information Questionnaire and the Self-Report Vocabulary Knowledge Scale (SRVMS). The Demographic Information Questionnaire was administered before the intervention to gather background information about the participants, including their age and gender. It also provided an overview of the study and included instructions on how to complete the form. To ensure anonymity, participants were not asked to provide their real names; instead, they were instructed to use a pseudonym and an assigned number. The SRVMS (check Appendix A) was administered both before and after the teaching intervention. This scale was designed to measure learners' vocabulary knowledge, focusing on their ability to understand and use the target words taught during the program. The same 35 vocabulary items used in instruction were also used as test items in the SRVMS. To ensure validity and reliability, the SRVMS was piloted with a similar group of 62 students prior to the main study, using the same target words. The pilot results indicated that the scale was reliable for measuring learners' vocabulary knowledge in this context. The pre-treatment administration served as a baseline for learners' initial vocabulary knowledge, while the post-treatment version allowed the researchers to assess vocabulary gains and evaluate the effectiveness of the instructional methods. The scoring system, adapted from Esit's (2007) thesis and subsequently modified for this study, was reviewed by a statistician to ensure its validity. The scoring system is described in Table 2.

**Table 2:** The scoring system of SRVMS.

**Score**	**Criteria**
1	Option A: Recognizes the word but does not understand its meaning
2	Option B: Understands the word's meaning but cannot use it in context.
3	Option C: Knows the word's meaning but cannot construct a sentence
4	Option D: Knows the meaning and constructs a grammatically and semantically correct sentence. Minor mistakes are acceptable

Scores range from 1 to 4, with higher scores indicating greater vocabulary mastery.

### 3.2 Target words and rationale for word selection

For this study, researchers selected 35 target vocabulary items from the *Active Learn* online readers' platform. These words were chosen from 12 books at the Gray and Blue levels, which are designed for learners at the participants' proficiency level. Importantly, these particular books had not been previously used in classroom instruction, and the students had no prior exposure to the selected words. the *Active Learn* platform was familiar to students as part of their regular learning environment, care was taken to ensure that the specific vocabulary items used in this study were new to them. Additionally, to isolate the effect of the instructional methods, the books themselves and their accompanying digital activities were not used during the intervention. The selected vocabulary was categorized according to grammatical type and semantic field, as shown in Table 3.

**Table 3:** Target words.

**Category**	**Words**	**Numbers**
Nouns	Telescope, pyramid, backpack, jungle, waterfall, helmet, castle, seahorse, compass, staircase, adventure, journey	12
Verbs	Notice, rescue, sprint, sketch, spin, whisper, explore, disguise, leap, navigate, discover	11
Adjectives	Bright, silent, narrow, heavy, dusty, shiny, smooth, fierce, muddy, brave, ancient, mysterious	12

### 3.3 Procedure

Before the study began, ethical approval was obtained from the affiliated university to ensure that all procedures followed established research guidelines, particularly because the participants were minors. Once approval was granted, the researchers contacted the participating language school and received verbal permission to carry out the study. Since all students were under the age of 18, parental consent forms were also sent out and collected in advance (see Appendix B).

The study followed a quasi-experimental design to explore how the order of presenting vocabulary focusing on meaning first vs. form first might affect young learners' ability to acquire new words. Two comparison groups were formed, and to ensure impartial group assignment, participants were randomly allocated by flipping a coin. Both groups were taught the same set of 35 vocabulary words over 12 weeks, with three 40-min lessons each week.

#### 3.3.1 Instruction in Comparison Group 1

Students in Comparison Group 1 were taught new words using this teaching technique which consists of five stages. Below is a sample lesson plan demonstrating how the target word *notice* is taught:

*Stage 1*: Contextualization: The instructors set up a scenario to engage the students and create a concept in their minds. For example, students are presented with a situation where Jane is walking and encounters a stone, leading to a potential fall. The researchers ask the students to think about why this happened.
*Stage 2*: Guessing and Explanation: The students attempt to guess the answer, and if they are unable to do so, the instructors provide the explanation. They explain that Jane didn't see the stone and didn't “notice” it. The researchers repeat the sentence with the target word included if necessary.
*Stage 3*: Introduction of the Written Form The researchers present the written form of the target word, “notice,” and ask the learners to repeat it.
*Stage 4*: Visual Differentiation: Students are presented with a series of pictures that illustrate situations where the word *notice* applies. For example: A person looking at a sign on the wall.
  Someone pointing out a detail in a crowded room. A person suddenly realizes a missing item in their bag. Learners are encouraged to create sentences by looking at the pictures and using the target word.
*Stage 5*: In the final stage of this teaching technique, instructors provide learners with sample sentences. Learners are then asked to copy these sentences into their notebooks and repeat them aloud. This activity helps students see the target words in context, practice their use, and improve their pronunciation. The sample sentences are She didn't notice that the wall was painted and They didn't notice the sign and brought food and drinks to the cinema.

#### 3.3.2 Instruction in Comparison Group 2

Students who are in Comparison Group 2 are taught new words by using this technique. In this type of instruction, learners are exposed to oral commands of the target words and the meanings of these words are presented after learners are fully engaged with the spoken form of the words. The teaching process adopted for this study is outlined in five stages below, illustrated using the example of the word *noticed*:

*Stage 1*: On this stage the researchers choose the target word and begin the lesson by informing the students that the specific word they will be learning is “*notice*.” They repeat the word multiple times to ensure that the learners hear and become familiar with its pronunciation.
*Stage 2*: The students are asked to repeat the target word themselves. Researchers listen to each students' pronunciation and provide corrections when necessary. The aim is to ensure that the learners accurately pronounce the word and develop a clear understanding of its spoken form.
*In Stage 3*, the researchers present sample sentences to the learners that contain the target word “notice.” These sentences are designed to provide contextual usage of the word and to further reinforce the learners' understanding and familiarity with its meaning and usage.

For instance, the researchers provide sentences such as “She didn't notice that the wall was painted” or “They didn't notice the sign and brought food and drinks to the cinema.” These sentences demonstrate different contexts in which the word “notice” can be used and help learners grasp its meaning in various situations. During this stage, the researchers encourage the learners to read these sentences aloud. By doing so, the learners actively engage with the sentences and practice their pronunciation skills. The researchers listen to the learners' pronunciation and provide corrections if necessary, ensuring that the learners accurately articulate the target word and the surrounding sentence. By incorporating sentence-level practice, learners not only enhance their understanding of the target word's meaning but also develop their ability to use it appropriately within a given context. This stage promotes both comprehension and production skills, allowing learners to grasp the nuances of the word and build confidence in using it effectively.

*In Stage 4*, after learners have been exposed to the target word “notice” through repetition, pronunciation practice, and encountering it in sample sentences, the researchers proceed to explain its meaning to the learners. Specifically, they focus on presenting the frequently used meaning of the word. For example, the researchers explain that “notice” means to become aware of something or to pay attention to something. They may provide additional clarification or examples to further illustrate the meaning. The goal is to ensure that learners understand the primary and commonly used sense of the word in different contexts. By explaining the meaning of the target word, learners gain a deeper understanding of its significance and how it is used in various situations. This stage helps consolidate their comprehension of the word and allows them to connect it to their existing knowledge and experiences. It is important to note that this stage focuses on comprehension rather than production. Learners are primarily developing their receptive skills, understanding how the word is used and its meaning in context. The subsequent stage will provide opportunities for learners to actively produce the word.

*In Stage 5*, learners are presented with pictures that are relevant to the target word “notice.” The pictures cover:

A person looking at a sign on the wall.

Someone pointing out a detail in a crowded room.

A person suddenly realizing a missing item in their bag.

The learners are encouraged to observe the pictures carefully and actively participate in the following discussion.

What do you notice in this picture? How can someone show that they noticed something?

## 4 Results

This section presents the key findings of the study, emphasizing the effects of different instructional approaches on vocabulary acquisition. The results were analyzed using IBM SPSS Statistics Version 30. Pre-test results indicated that both groups began at a comparable level, confirming the absence of significant differences in vocabulary knowledge prior to the intervention. However, post-test results demonstrated that students in Comparison Group 1, who received meaning-first instruction, achieved significantly greater gains than those in Comparison Group 2, who followed a form-first approach. These findings suggest that introducing vocabulary through its meaning may enhance young learners' ability to retain and apply new words more effectively. The subsequent sections offer a detailed analysis of these results.

**Sub research question (a) Is there any disparity in the pre-test results between both groups?**

To ensure both groups started from a similar point, a pre-test was administered before the intervention. The results showed that Group 1 had a mean score of 39.06 (SD = 4.015), while Group 2 had a mean of 38.14 (SD = 2.965). Although there was a slight difference between the groups, it was not statistically significant (*p* > 0.05), indicating that the students' vocabulary knowledge was roughly equivalent at the beginning of the study. This is presented in Table 4.

**Table 4:** Pre-test results of Group 1 and Group 2.

**Participants**		***N***	**M**	**M_2_-M_1_**	**SD**	***P***	**Level of significance**
Groups	Group 1	27	39.06	0.916	4.015	0.139	*p* > 0.05
	Group 2	28	38.14		2.965		

**Figure 1:**
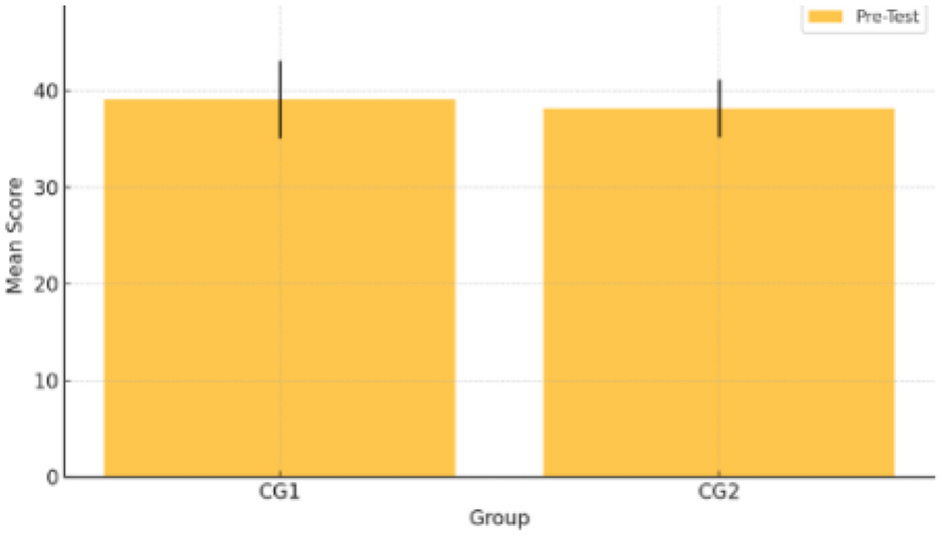
Pre-test results of Comparison Group 1 and Comparison Group 2.

As shown in Figure 1, the pre-test scores of both groups were visually comparable, further supporting the statistical findings of baseline equivalence.

To evaluate the difference between the two groups in the pre-test, the effect size was calculated using Cohen's d. The pooled standard deviation was approximately 3.54, and the resulting effect size was 0.26, which indicates a small effect. This suggests that the two groups were relatively similar in their performance at the baseline, as expected in the pre-test, where homogeneity between groups is essential before applying the intervention.

**Sub research question (b) Is there any disparity in the post-test results between both groups?**

According to the data presented in Table 5, the mean value of the post-test scores for Comparison Group 1 (meaning-first instruction) was 187.12, while for Comparison Group 2 (form-first instruction) it was 114.00. The results clearly indicated a statistically significant difference between the two groups. The calculated significance value was 0.00, which was lower than the predetermined threshold of 0.05 (*p* < 0.05). Therefore, it could be concluded that students in Comparison Group 1 achieved higher scores compared to students in Comparison Group 2.

**Table 5:** Post-test results of Comparison Group 1 and Comparison Group 2.

**Participants**		***N***	**M**	**M_2_-M_1_**	**SD**	***P***	**Level of significance**
Groups	CG1 1	29	187.12	73.118	16.816	0.000	*p* < 0.05
	CG2 2	28	114.00		30.884		

**The main research question:**

1) Does the meaning first-first approach or form-first triggered approach result in differences in vocabulary retention?

According to the data presented in Table 5, the mean post-test score for Comparison Group 1 (meaning-first instruction) was 187.12, while the mean for Comparison Group 2 (form-first instruction) was 114.00. This large difference in post-test means indicates that students in the meaning-first group significantly outperformed their peers in the form-first group. The independent-samples *t*-test revealed that this difference was statistically significant (*p* = 0.000), which is below the commonly accepted threshold of 0.05, confirming the effectiveness of the meaning-first approach in enhancing vocabulary acquisition. To further illustrate the impact of the instructional methods on students' vocabulary acquisition, Table 5 presents a comparison of post-test results between the two groups. This table highlights the differences in mean scores, standard deviations, and statistical significance levels, offering a more detailed understanding of how each group responded to the intervention. The data provide empirical support for the claim that meaning-first instruction results in greater vocabulary gains than form-first instruction.

**Figure 2:**
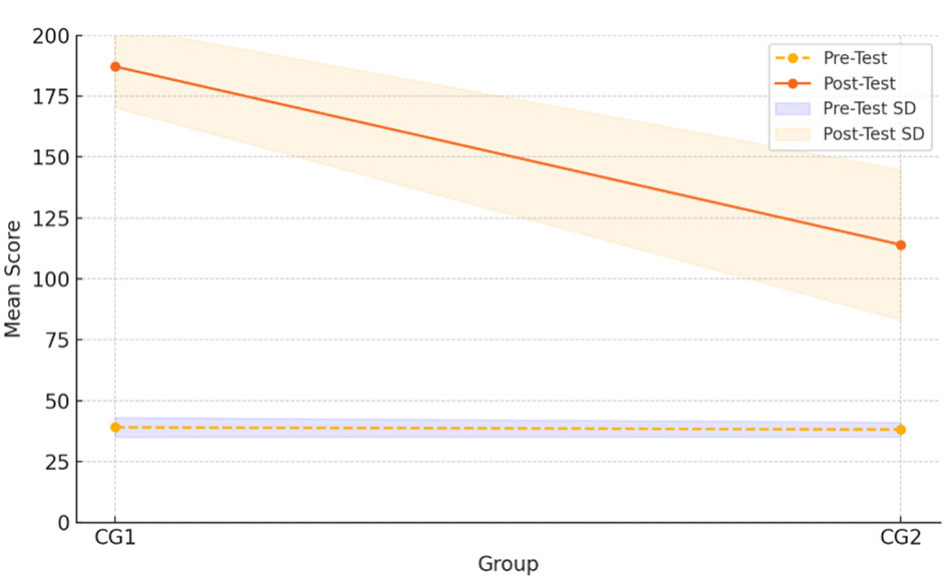
Comparison of pre-test and post-test results.

Table 6 presents the post-test results of both groups, comparing their vocabulary gains following the instructional intervention. While both the meaning-first (CG1) and form-first (CG2) groups showed improvement, students in the meaning-first group achieved substantially higher mean scores (M = 187.12, SD = 16.82) compared to the form-first group (M = 114.00, SD = 30.88). The independent-samples *t*-test confirmed that this difference was statistically significant, *t*_(55)_ = 4.89, *p* < 0.001, with a large effect size (Cohen's *d* = 1.37). This is also supported on Figure 2.

**Table 6:** Post test results of Comparison Group 1 and 2.

**Group**	***N***	**M**	**SD**	***T*(df)**	***p***	**Cohen's *d***
CG1 (Meaning-first)	29	187.12	16.82	–	–	5.59
CG2 (Form-first)	28	114.00	30.88	–	–	4.82
Between groups	–	–	–	4.89	< 0.001	1.37

As presented in Figure 2, students in the meaning-first group showed markedly greater gains between the pre- and post-test compared to the form-first group. These results support the conclusion that meaning-first instruction was more effective in enhancing vocabulary acquisition among young learners. it could be concluded that students in Comparison Group 1 achieved higher grades compared to students in Comparison Group 2.

## 5 Discussion

Before addressing the main research question, it is necessary to evaluate the comparability of the two groups at the outset of the study. The first sub-research question explored whether any significant differences existed between learners' vocabulary knowledge prior to the intervention. Analysis of the pre-test scores using the Self-Report Vocabulary Knowledge Scale (SRVMS) revealed no statistically significant difference between the groups (*p* >0.05), confirming baseline equivalence. This homogeneity is essential for the internal validity of the study, as it ensures that observed differences in outcomes can be attributed to the instructional intervention rather than to pre-existing disparities. Similar results were reported in previous quasi-experimental studies (Esit, 2007; Basal et al., 2016), further supporting the reliability of using intact classes in educational settings when randomization is not feasible.

The second sub-research question examined the effectiveness of meaning-first vs. form-first instruction in enhancing vocabulary acquisition. The findings demonstrated that although both instructional methods led to significant improvement, students in the meaning-first group exhibited significantly greater gains in both vocabulary knowledge and sentence-level usage. These results underscore the pedagogical value of introducing words through meaning-rich contexts rather than through decontextualized form.

These findings provide important contributions to the ongoing debate in vocabulary instruction. While Nation (2013) underscores the value of inferring meaning through context, the present study suggests that direct and early exposure to meaning, particularly in the context of young bilingual learners, may produce more robust gains. Contrary to studies advocating form-first sequencing (e.g., Iamsirirak, 2017), our results indicate that beginning with form may limit depth of processing and impose a higher cognitive load on working memory, especially for younger learners navigating two linguistic systems.

This interpretation is supported by cognitive theories such as Baddeley's (2003) working memory model and Craik and Lockhart's (1972) depth of processing theory. Meaning-first instruction likely reduces extraneous cognitive load by anchoring new words in prior conceptual knowledge, thus facilitating long-term storage in episodic memory. Moreover, according to dual coding theory (Paivio, 1991), the use of visual aids and contextual cues in meaning-first instruction likely activated multiple representational systems, reinforcing recall and application. These results also align with the psycholinguistic perspective that semantic processing plays a pivotal role in lexical retention (Jenkins and Dixon, 1983; Nagy and Anderson, 1984). Learners in the meaning-first group not only acquired the words but also used them more meaningfully in context, indicating deeper mental integration. The large effect sizes observed in this study further suggest that meaning-first instruction offers not just statistical, but also practically significant benefits for vocabulary learning in multilingual primary classrooms.

Importantly, this study extends the work of researchers such as Laufer (2005) by providing empirical evidence that rich, meaning-focused instruction is especially advantageous for young bilingual learners, a group often underrepresented in experimental vocabulary research. It also contributes to second language acquisition literature by reinforcing the importance of conceptual priming in early vocabulary instruction (Neely, 1977; Milton, 2010).

Taken together, the findings challenge traditional form-first paradigms and advocate for a reconceptualization of vocabulary pedagogy in primary and bilingual education. While Laufer (2005) rightfully notes that comprehensible input alone may not ensure acquisition, this study demonstrates that when meaning-first instruction is paired with structured, scaffolded tasks, it significantly enhances both vocabulary retention and meaningful application.

## 6 Conclusion

This study offers important insights into how vocabulary should be taught in today's classrooms, particularly for young learners navigating more than one language. Traditional vocabulary instruction often begins with isolated drills, word lists, and a focus on form how a word sounds, how it's spelled, or where it fits grammatically. While these elements have their place, our findings suggest that this method may not be the most effective or engaging way for children to truly understand and remember new words.

Instead, the results of this study show that when students first encounter words in meaningful contexts through stories, real-life examples, or visual scenes they are not only more likely to retain those words, but they also use them more confidently and naturally. The meaning-first approach appears to support the way children process and internalize language, helping them move beyond memorization to actual communication. This is especially significant for bilingual or multilingual learners, who may already be balancing multiple language systems in their everyday lives.

By comparing two instructional approaches in real classroom settings, this research adds a practical and research-based perspective to the ongoing discussions in second language acquisition. It supports the idea that language learning is not just about input, but about how that input is delivered and whether it invites learners to think, connect, and use language meaningfully. These findings are not just statistical; they reflect real shifts in how children experience language, interact with new words, and grow in their ability to express themselves.

Educators, curriculum developers, and policy makers should take note of these outcomes. Vocabulary instruction that begins with meaning rather than form can help build stronger foundations in language and open the door to deeper learning across all subjects. Integrating context-rich vocabulary practices into everyday lessons doesn't require an overhaul of the curriculum; rather, it calls for a shift in mindset one that places learners' cognitive and emotional engagement at the heart of teaching.

As education systems continue to evolve in response to diverse classroom needs, studies like this one highlight the importance of aligning pedagogy with how children actually learn. Looking ahead, future research could explore how meaning-based instruction impacts long-term fluency, reading comprehension, and academic confidence. But what this study already makes clear is that when children are given the chance to connect with words in a way that feels real and relevant, they do more than learn they thrive.

## 7 Limitations of the study

Similar to all classroom-based research, this study has its limitations. First, it focused only on Grade 4 students, which means the findings may not fully reflect how older or younger learners respond to meaning-first instruction. Future research could explore whether similar patterns hold across different age groups. Second, the study took place over a relatively short period. While the gains were clear, a longer-term investigation would be valuable to see how well students retain and apply new vocabulary over time. Lastly, although the results suggest meaningful cognitive differences between the two instructional approaches, the study did not include neuroscientific tools such as eye-tracking or brain imaging. Including such methods in future work could offer deeper insight into how learners process words at a neurological level. Addressing these limitations in future studies would strengthen the evidence base and contribute to more informed instructional practices. Finally, while this study was carried out in a specific bilingual school context in Cyprus, the results might not reflect what would happen in other bilingual or multilingual classrooms. Future research could explore how these findings apply in different educational settings, language combinations, or cultural environments to better understand how broadly the approach can be used.

## Data Availability

The original contributions presented in the study are included in the article/supplementary material, further inquiries can be directed to the corresponding author.

## Appendix A

### Vocabulary knowledge scale

_____________________________________________________

Category Meaning of category (Esit, 2007) _____________________________________________________

I don't remember having seen this word before
I have seen this word before, but I don't know what it means.
I have seen this word before and I think it means________________(synonym or translation)
I know this word. It means________________(synonyms or translation) and I can use the word in a sentence.

_____________________________________________________

## Appendix B

### Vocabulary teaching: an experimental study

**                                  Parental consent form**

I confirm that I ________________________________ am the parent/legal guardian of ___________________________________________.

I hereby consent to the above child participating in experimental research study in line with the Code of Ethics & Good Practice for Children's Sport. I have provided contact details below I confirm that all details are correct and I am able to give parental consent for my child to participate in all activities related to the research.

Name:_________________________________________

Signature _____________________________________

Contact Details

Name of Child__________________________________________

Address_______________________________________________

_______________________________________________

Parent's Mobile Phone No. ________________________________

Emergency Contact No. (1)________________________________

Emergency Contact No. (2) ________________________________

Please also include all medical details that might be relevant in dealing in with your child in a safe manner, such as allergies, medication, special needs, etc.

___________________________________________________

Photographic & Video Consent

I consent/do not consent to the below mentioned child being included in any photographic or video material, in any publications/websites/social network applications which may be used for the purpose of documenting and highlighting their involvement in the study.

Name: ___________________________________________

Age: ________

Signature: _________________________________________

Date: _______________

Print Name: ________________________________________

State Relationship to child: ____________________________

Phone No. _________________
